# Public health framing of firearm violence on local television news in Philadelphia, PA, USA: a quantitative content analysis

**DOI:** 10.1186/s12889-024-18718-0

**Published:** 2024-05-03

**Authors:** Jessica H. Beard, Shannon Trombley, Tia Walker, Leah Roberts, Laura Partain, Jim MacMillan, Jennifer Midberry

**Affiliations:** 1 Department of Surgery, Division of Trauma Surgery and Surgical Critical Care, Lewis Katz School of Medicine, Temple University, 3401 N. Broad St, 4th Floor, Zone C, Philadelphia, PA 19140 USA; 2 Lewis Katz School of Medicine, Temple University, Philadelphia, PA USA; 3 Department of Epidemiology, Mailman School of Public Health, Columbia University, New York, NY USA; 4 School of Communication, Ohio State University, Columbus, OH USA; 5 Philadelphia Center for Gun Violence Reporting, Philadelphia, PA USA; 6 Department of Journalism and Communication, Lehigh University, Bethlehem, PA USA

**Keywords:** Firearm violence, Violence prevention, News framing, Health communication

## Abstract

**Background:**

Firearm violence is an intensifying public health problem in the United States. News reports shape the way the public and policy makers understand and respond to health threats, including firearm violence. To better understand how firearm violence is communicated to the public, we aimed to determine the extent to which firearm violence is framed as a public health problem on television news and to measure harmful news content as identified by firearm-injured people.

**Methods:**

This is a quantitative content analysis of Philadelphia local television news stories about firearm violence using a database of 7,497 clips. We compiled a stratified sample of clips aired on two randomly selected days/month from January-June 2021 from the database (*n* = 192 clips). We created a codebook to measure public health frame elements and to assign a harmful content score for each story and then coded the clips. Characteristics of stories containing episodic frames that focus on single shooting events were compared to clips with thematic frames that include broader social context for violence.

**Results:**

Most clips employed episodic frames (79.2%), presented law enforcement officials as primary narrators (50.5%), and included police imagery (79.2%). A total of 433 firearm-injured people were mentioned, with a mean of 2.8 individuals shot included in each story. Most of the firearm-injured people featured in the clips (67.4%) had no personal information presented apart from age and/or gender. The majority of clips (84.4%) contained at least one harmful content element. The mean harmful content score/clip was 2.6. Public health frame elements, including epidemiologic context, root causes, public health narrators and visuals, and solutions were missing from most clips. Thematic stories contained significantly more public health frame elements and less harmful content compared to episodic stories.

**Conclusions:**

Local television news produces limited public health coverage of firearm violence, and harmful content is common. This reporting likely compounds trauma experienced by firearm-injured people and could impede support for effective public health responses to firearm violence. Journalists should work to minimize harmful news content and adopt a public health approach to reporting on firearm violence.

**Supplementary Information:**

The online version contains supplementary material available at 10.1186/s12889-024-18718-0.

## Background

Community firearm violence, defined as fatal (e.g. firearm homicide) and non-fatal shootings that result from interpersonal violence, is an increasing threat to public health in the United States (US) [1]. In 2021, 20,996 people died from firearm homicide in the US, the highest recorded number of deaths since 1993 [2]. For each firearm homicide, there are at least two people who are non-fatally shot [3]. People who survive firearm injury often live with significant physical and psychological disability, and exposure to community firearm violence has far reaching community-level and societal consequences beyond individual gunshot wounds [4].

News media, including television, radio, print, and online platforms, play an influential role in shaping how the public and policy makers understand and respond to health threats, including firearm violence [5–10]. People with little to no direct experience with community firearm violence likely draw on information from news and other types of media to form their understanding of the issue. Therefore, the manner in which news stories depict a shooting incident can influence the attitudes of news audiences towards this public health problem [11–13]. This process can be understood through framing theory, which posits that news reporting inevitably emphasizes certain aspects of a news event at the expense of others, resulting in specific narratives that are likely to be adopted by news audiences and shape their views [11–13]. In other words, journalists’ decisions about what to cover, what details to include or exclude, which voices to highlight or pass over, and how to arrange the information will all affect how salient these elements are to news consumers [11–13].

One reason that community firearm violence is not more widely recognized as a public health problem may be that violence is rarely presented, or framed as a public health problem in US news [5, 7–9, 14]. Instead, news reports depict violence almost exclusively as a crime issue [8, 15–18]. For decades, crime stories have been a central feature of US news, dominating headlines and leading newscasts, because they are considered more newsworthy and attention-grabbing than other types of events [17, 18]. Research indicates that news coverage of crime and violence relies almost entirely on police sources and rarely includes the perspectives of victims [8, 15, 16, 19]. Additionally, news stories typically present violence to the public using *episodic framing*, when a story focuses on a single incident in isolation, as opposed to employing *thematic framing*, in which a news report explores the broader social and structural context in which violence occurs [5, 8, 9, 14]. *Episodic crime reporting *is the intersection of news framing that is episodic, defines violence as a crime issue, and privileges police narrators above other perspectives. Studies indicate that episodic crime reports on violence can lead audiences to blame victims, reinforce racist stereotypes about the people and places impacted, suggest an unfounded efficacy to policing as a means to prevent violence, and in turn, undermine effective public health responses [5, 7–9, 14, 16, 20–22]. When law enforcement officials are regularly presented as primary sources in stories on violence, news audiences have been shown to adopt normative news narratives, which prioritize expert opinions and eyewitness testimony of those beholden to state interests (e.g. police, politicians) over those most affected by the news event [11–13, 16, 21]. For decades, scholars have urged journalists to stop framing firearm violence predominantly as a crime issue and start covering it as a public health issue, but these calls have largely been unheeded [8, 14, 23, 24].

A recent study revealed that these patterns in how firearm violence is framed in most US news stories can have detrimental effects not just on general audiences but also on the survivors of firearm violence themselves [25]. In a qualitative interview study, firearm-injured people relayed that episodic crime narratives in news reports of their shootings that neglected their personal perspectives felt dehumanizing and compounded their trauma [25]. Firearm-injured people in this study also described how other harmful news elements, including graphic content, inaccuracies, and mention of treating hospital resulted in distress, harm to their reputation, and threats to personal safety [25].

While the literature on the negative impacts of episodic crime framing of violence is robust, examinations of the content of news reporting on firearm violence specifically are relatively few [6, 7, 9, 10]. To date, there have been no studies which measure harmful media reporting on firearm violence, and to our knowledge, the framing of firearm violence on television news has not been examined since the 1990s [8]. There is a critical need to fill these gaps in knowledge to better understand how firearm violence is represented to the public and, in turn, how news might be shaping public opinion during this significant surge in community firearm violence in the US [26–28]. Therefore, the aims of this quantitative content analysis of local television news reports on firearm violence in Philadelphia are: (1) To determine the extent to which firearm violence is framed as a public health problem and (2) To quantify harmful news content on firearm violence as identified by firearm-injured people.

## Methods

### Setting and context

Polling indicates that more people in the US get their news from television than from other legacy (e.g. radio and print) sources [29]. Although a majority of consumers access news online, most Internet news sites repackage content from their own offline platforms or aggregate stories from legacy sources [30]. Therefore, television news is the modality with the largest reach that also creates entirely original reporting, making it a logical place to start an investigation of the framing of firearm violence in local news. Philadelphia, the location of this study, is the birthplace of Eyewitness News (1965) and Action News (1970), two local television newscasts that pioneered reporting approaches that have been critiqued for their production and circulation of negative narratives about Black communities [31]. These Philadelphia newscasts’ stereotypical framing of Black people, crime, and violence sustained structural racism throughout the US, as broadcasts around the country replicated their models [31]. Additionally, the epidemic of community firearm violence in Philadelphia is representative of cities across the US, with increasing rates of shootings since the onset of the Coronavirus-19 pandemic (COVID-19), disproportionate impact on young people and Black people, and place-based risk that has been linked to historic racism [26–28, 32]. For these reasons, Philadelphia is an ideal location to perform this research study.

### Design and sample

This study utilized quantitative content analysis, an approach that identifies trends in media content [33, 34]. The study sample was drawn from a database created by members of the research team of television news stories about firearm violence broadcast by the four major local Philadelphia stations in 2021. This database includes every local and national segment pertaining to firearm violence that aired between January 1, 2021 and December 31, 2021 on 6 ABC (WPVI-TV), NBC 10 (WCAU), CBS 3 (KYW-TV), and FOX 29 (WTXF-TV) during 6:00 a.m., 6:00 p.m., and 11:00 p.m. newscasts. We collected the video clips using TVEyes, a subscription recording program [35]. A comprehensive list of 37 search terms was developed to perform daily keyword searches in TVEyes. As an example, some of the keywords used were: “gun violence,” “shooting,” “homicide,” and “firearm” (See Additional file 1 for a full list of search terms). Research team members reviewed the search results manually, discarded segments unrelated to firearm violence, and recorded relevant videos and corresponding textual transcripts. The completed database includes 7,497 individual stories.

For the present study, a stratified sample of clips from January through June 2021 was compiled from the database. In accordance with best sampling practices for a quantitative content analysis of television news, we used a random number generator to select two days from each month during the study period for analysis [33]. All of the clips collected on the randomly-selected 12 days comprise the current dataset, including 192 video clips that amount to over 3.7 h of content.

### Procedures, coding instrument, and variables

Informed by the existing literature on news content and framing theory, JHB and JM developed the initial coding instrument and conducted coder training with co-authors ST and TW [5, 6, 8, 9, 12–14, 25]. During several rounds of training, the coding instrument was revised to modify variable coding parameters and clarify coding instructions as needed. Video clips collected from TVEyes outside the study period using the same keywords and procedures as the content in the database were used for coding training. The visual, verbal, and textual elements were all taken into account during coding, as each of these elements conveys specific information to audiences [33–36].

We calculated several measures of intercoder reliability (ICR): simple percent agreement, Krippendorf’s and Gwet’s AC_1_. For several variables, the Krippendorf’s alpha scores were low in spite of high percent agreement numbers. In these instances, the coded answers fell largely into just one category choice, therefore, the Gwet’s AC_1_results were prioritized, as recommended by Lacy and colleagues [37]. Coders worked with practice material until they reached ICR scores of at least 0.7 using Krippendorf’s alpha and Gwet’s AC_1._ Then they coded the same, overlapping 10% of the study sample clips, reaching acceptable levels of ICR. These ICR scores are reported below for key variables, and Additional file 2 lists the individual ICR scores for each coded variable as well as the operationalization of all the variables. After coding the same 10% of the sample, each coder then coded half of the remaining video clips individually.

Two variables did not require ICR calculations. *Clip length* was taken directly from each video’s time stamps and reported in minutes and seconds. *Story location* was open coded, based on information in the chyrons and the audio. Locations were then organized into four categories: Philadelphia, local (outside Philadelphia), national, multiple locations.

Four variables were coded to quantify specific narrative elements. *Framing* (97.8% agreement, α = 0.921), determined whether clips used episodic or thematic framing. Clips were coded as *episodic framing* if the majority of the time focused on a specific shooting event(s), and they were coded as *thematic framing*if most of the clip discussed firearm violence more broadly, including social context, epidemiological trends, root causes, and/or solutions [8, 23]. *Narrators* (88.0% agreement, α = 0.841) documented everyone interviewed or shown speaking in a story. The *primary narrator* variable identified the person seen/heard speaking for the longest amount of time in a clip (86.7% agreement, α = 0.813). *Police attribution* (93.3% agreement, α = 0.867) was coded to record each instance of a journalist identifying police as the source of information for a story (e.g.: “law enforcement tells us,” “according to police,” “authorities say”). Additional file 3 provides several exemplars of text from television news reports included in this study, including (1) Episodic report with police attribution; (2) Episodic report with law enforcement representative as primary narrator; and (3) Thematic report with community members as narrators.

Eight variables were used to determine how often potentially harmful content elements were included in the sample. Firearm-injured people in the previously mentioned qualitative study identified these elements as harmful [25]. Seven variables were coded (yes/no) to indicate whether the harmful content elements were present in a clip: *visual of crime scene*, *not a follow-up story, number of gunshot wounds*, *clinical condition of firearm-injured person, relationship between firearm-injured person and shooter, name of treating hospital, video or audio of shooting.* The variable *not a follow-up story* identified whether the story represented initial breaking news about the shooting event or covered the subsequent impact of firearm violence. A follow-up story was defined as one that takes place > 24 h after the shooting. *Not a follow-up story* also had options for coding the response as “Unknown” if the shooting date and time was unclear or “Not applicable” if the clip did not cover a specific shooting event. The variable *not a follow-up story* yielded 75.6% agreement and AC_1_= 0.696, which falls just short of the 0.7 threshold for acceptable ICR [25]. As this is a nascent area of interdisciplinary work, we report the results for *not a follow-up story*, with the caveat that they should be interpreted as tentative. The ICR scores for the other six variables ranged from 82.2% to 100% agreement and AC_1_ = 0.802 to 1. The eighth harmful content variable was *only narrator is law enforcement*, which was calculated during the analysis by tallying the instances in the *narrators variable* where 1) a journalist was the only narrator and attributed police sources and 2) a law enforcement representative was the only person interviewed. A harmful content score for each clip was calculated by summing the number of harmful content elements present in each clip. Each element contributed one point, for a maximum score of eight.

The presence or absence of additional visuals was coded using binary yes/no answers. These variables, *police imagery, firearm-injured person and/or family, community event, mugshot, political press conference*, ranged from 91.1% to 100% agreement and AC_1_ = 0.894 to 1.

Six variables noted characteristics of firearm-injured people: *number of people injured, specific firearm-injured person identified, fatality, age, race/ethnicity, and gender*. ICR scores ranged from 95.6% to 100% agreement and α = 0.912 to 1. It would have been ideal to have a variable for the etiology of firearm injury. However, this was not possible, because most of the coverage did not include enough context to accurately assess etiology. We also recorded several other variables relating to additional personal information of firearm-injured people: *photograph, name, family information, occupation, educational level, criminal record*. ICR values for all of these was 100% agreement and α = 1.

**Fig. 1:**
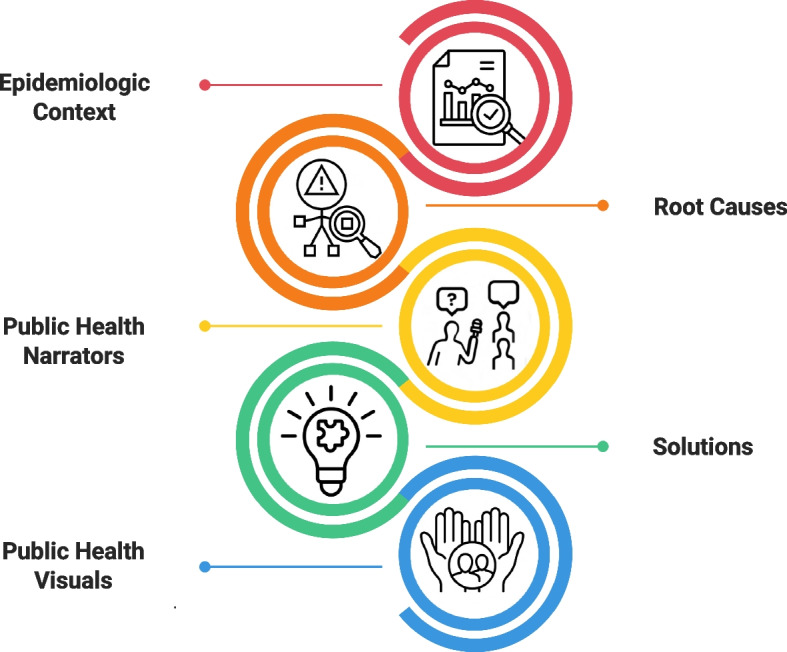
Elements of a public health frame in news reports on firearm violence

Drawing on work by Dorfman and colleagues, we posited that the elements of a public health frame about firearm violence would include: (1) epidemiologic context (through data or trends); (2) root causes; (3) public health narrators (e.g. public health professionals, community representatives, politicians, and firearm-injured people and/or loved ones); and (4) public health visuals (e.g. community events, interviews with community representatives, or firearm-injured people and/or loved ones) and (5) solutions [8, 20, 23, 24]. Figure 1 is a visual depiction of news elements in stories on firearm violence that constitute a public health frame. To assess whether these elements were present in the sample of television news clips analyzed, we coded for the presence or absence of several additional variables: *epidemiological context, root causes, public health root causes, solutions, specific solutions, public health solutions, emphasis on prevention, includes word “prevent,”* and *resources offered*. ICR scores ranged from 86.7% to 100% agreement and AC_1_ = 0.841 to 1.

### Analysis

Data were analyzed using descriptive and comparative statistical methods. Continuous outcomes are presented as means with standard deviations while nominal outcomes are presented as numbers with percentages. Characteristics of episodic and thematic framed stories were compared using used two-sample t-tests for the continuous variables and Chi-square tests or Fisher’s exact tests as appropriate for the categorical variables. A *p*-value < 0.05 was considered statistically significant. The data were analyzed using IBM SPSS version 27 and R Version 4.3.1.

## Results

Descriptive characteristics of the television news stories are presented in Table 1. Of the 192 clips coded in this study, the majority (68.2%) were local stories of firearm violence in Philadelphia. Nearly 80% of the stories employed episodic framing, and the most common primary narrator apart from the journalist was a law enforcement representative (21.4%). In clips where the journalists were the only narrators featured, 65.1% included police attribution, indicating that police are the predominant source of information in the majority of stories (50.5%) on firearm violence on local television news in Philadelphia. No segments included a health or public health professional or a firearm-injured person as the primary narrator. In fact, no clips featured either of these stakeholders at all, even as secondary narrators. Most of the stories included some police and/or crime scene imagery, and 14 clips (7.3%) included video or audio depiction of the shooting event.

**Table 1:** Descriptive characteristics of Philadelphia television news stories on firearm violence, January—June 2021

	Clip Characteristics, *n* = 192
Mean clip length in minutes: seconds ± SD, (range)	1:09 ± 1:01 (0:11–6:04)
Location of the story, n (%)	
Philadelphia	131 (68.2)
Local, outside Philadelphia	10 (5.2)
National, outside Philadelphia	45 (23.4)
Multiple locations	6 (3.1)
Framing, n (%)	
Episodic	152 (79.2)
Thematic	40 (20.8)
Primary Narrator, n (%)	
Journalist	86 (44.8)
Law enforcement representative	41 (21.4)
Community member	25 (13.0)
Politician	16 (8.3)
Family, friend, and/or neighbor of firearm-injured person	14 (7.3)
Lawyer	3 (1.6)
Unidentified person	3 (1.6)
Bystander and/or witness	2 (1.0)
Other	2 (1.0)
Firearm-injured person	0 (0)
Health and/or public health professional	0 (0)
Journalist is primary narrator with police attribution, n (%)	56 (65.1)
Visuals, n (%)^a^	
Police imagery	152 (79.2)
Crime scene	127 (66.1)
Photograph and/or interview with firearm-injured person and/or family	34 (17.7)
Community event	34 (17.7)
Mugshot of alleged perpetrator	24 (12.5)
Political press conference	21 (10.9)
Video and/or audio of shooting	14 (7.3)
Contains harmful content, n (%)	162 (84.4)
Mean harmful content score ± SD, (range)	2.6 ± 1.8 (0–7)
Specific harmful content elements, n (%):	
Visual depiction of crime scene	127 (66.1)
Story is not a follow-up	100 (52.1)
Only narrator is law enforcement	76 (39.6)
Number of gunshot wounds	75 (39.1)
Clinical condition of firearm-injured person	57 (29.7)
Relationship between firearm-injured person and shooter	25 (13.0)
Name of treating hospital	17 (8.9)
Video or audio depiction of shooting	14 (7.3)

*SD* Standard deviation

^a^Each clip may contain multiple visual elements

The majority of stories (84.4%) contained at least one harmful element of news content, and the mean harmful content score per clip was 2.6. The most common harmful content elements were visual depiction of the crime scene, lack of follow-up story, no narrators apart from law enforcement, and clinical information about the firearm-injured person (Table 1).

More than 80% of the stories mentioned a specific firearm-injured person (*n* = 154 clips). Information presented about firearm-injured people in television news stories is summarized in Table 2. In the 192 clips, there were a total of 433 firearm-injured people mentioned, with a mean of 2.8 individuals shot included in each story. Basic demographic information on age, gender, and race was missing for the majority of firearm-injured people mentioned in the segments, and 67.4% of the firearm-injured people included had no personal information presented apart from age and/or gender.

**Table 2:** Characteristics of firearm-injured people reported in Philadelphia television news stories on firearm violence, January—June 2021

	Characteristics of firearm-injured people (*n* = 433)
Mean number of firearm-injured people mentioned per story ± SD, (range)	2.8 ± 4.5 (1–49)
Mean age in years ± SD	25.6 ± 13.0
Firearm-injured person is a child (< 18 years), n (%)	
Yes	52 (12.0)
No	104 (24.0)
No age provided	277 (64.0)
Shooting fatal	
Yes	244 (56.4)
No	173 (40.0)
Unknown/Unclear from report	16 (3.7)
Race/ethnicity	
White	1 (0.2)
Hispanic	2 (0.5)
Black	24 (5.5)
Asian	43 (9.9)
Mulitracial	2 (0.5)
Unknown/Unclear from report	361 (83.4)
Gender	
Male	165 (38.1)
Female	61 (14.1)
Non-binary	0 (0.0)
Unknown/Unclear from report	207 (47.8)
Personal information provided^a^	
Photograph	84 (19.4)
Name	68 (15.7)
Family information	57 (13.2)
Occupation	23 (5.3)
Educational level	4 (0.9)
Criminal record	4 (0.9)
Mugshot	0 (0.0)
None	292 (67.4)

*SD* Standard deviation

^a^Personal information apart from age and/or gender

As shown in Table 3, the key elements of a public health frame were absent in the majority of Philadelphia television news stories on firearm violence. For example, data and epidemiologic trends were rarely used to contextualize firearm violence, root causes were mentioned in only 10.9% of segments, public health solutions were rarely discussed (10.4%), the word “prevent” was present in only 5 stories (2.6%), and resources were offered in just 4 clips (2.1%).

**Table 3:** Elements of a public health frame in Philadelphia television news stories on firearm violence, January—June 2021

	Clip Characteristics, *n* = 192
Epidemiologic context, n (%)	
Data or epidemiologic trends	33 (17.2)
Root Causes, n (%)	
Root causes presented	21 (10.9)
Public health root causes	15 (7.8)
Public Health Narrators, n (%)	
Community member	25 (13.0)
Politician	16 (8.3)
Family, friend, and/or neighbor of firearm-injured person	14 (7.3)
Firearm-injured person	0 (0)
Health and/or public health professional	0 (0)
Public Health Visuals, n (%)^a^	
Photograph and/or interview with firearm-injured person and/or family	34 (17.7)
Community event	34 (17.7)
Solutions, n (%)	
Mention of prevention, mitigation, and/or solutions	40 (20.8)
Public health solutions discussed	20 (10.4)
Includes the word “prevent”	5 (2.6)
Resources offered	4 (2.1)

^a^Each clip may contain multiple visual elements

Compared to stories with an episodic frame, stories with a thematic frame were less likely to contain harmful content elements identified by firearm-injured people, as indicated in Table 4. Thematic stories were significantly more likely to contain elements of a public health frame, including data and epidemiologic trends, discussion of root causes and solutions, narrators apart from law enforcement, and public health visuals. There was no difference in the mean clip length between episodic and thematic stories on firearm violence on television news in Philadelphia.

**Table 4:** Characteristics of episodic compared to thematic Philadelphia television news stories on firearm violence, January—June 2021

	Episodic, *n* = 152	Thematic, *n* = 40	*P*-value
Mean clip length in minutes: seconds ± SD, (range)	1:08 ± 1:03 (0:11–6:04)	1:12 ± 0:52 (0:15–3:49)	0.67
Primary Narrator, n (%)			
Journalist	81 (53.3)	5 (12.5)	< 0.001
Law enforcement	34 (22.4)	7 (17.5)	0.50
Community member	10 (6.6)	15 (37.5)	< 0.001
Politician	5 (3.3)	11 (27.5)	< 0.001
Family, friend, and/or neighbor of firearm-injured person	13 (8.6)	1 (2.5)	0.31
Lawyer	3 (2.0)	0 (0.0)	> 0.999
Unidentified person	2 (1.3)	1 (2.5)	0.51
Bystander and/or witness	2 (1.3)	0 (0.0)	> 0.999
Other	2 (1.3)	0 (0.0)	> 0.999
Firearm-injured person	0 (0.0)	0 (0.0)	> 0.999
Health and/or public health professional	0 (0.0)	0 (0.0)	> 0.999
Journalist is primary narrator with police attribution, n (%)	56 (36.8)	0 (0.0)	< 0.001
Community representative is shown or heard speaking, n (%)	21 (13.8)	19 (47.5)	< 0.001
Politician is shown or heard speaking, n (%)	9 (5.9)	14 (35.0)	< 0.001
Visuals, n (%)			
Police imagery	128 (84.2)	24 (60.0)	< 0.001
Crime scene	112 (73.7)	15 (37.5)	< 0.001
Photo/interview with firearm-injured person/family	29 (19.1)	5 (12.5)	0.33
Mugshot of Perpetrator	21 (13.8)	3 (7.5)	0.28
Community Events	19 (12.5)	15 (37.5)	< 0.001
Video/Audio of Shooting	12 (7.9)	2 (5.0)	0.74
Political Press Conference	7 (4.6)	14 (35.0)	< 0.001
Contains harmful content, n (%)	142 (93.4)	20 (50.0)	< 0.001
Mean harmful content score + SD, (range)	3.0 + 1.7 (0–7)	0.8 ± 0.9 (0–3)	< 0.001
Specific harmful content elements, n (%):			
Visual depiction of crime scene	112 (73.7)	15 (37.5)	< 0.001
Story is not a follow-up	97 (63.8)	3 (7.5)	< 0.001
Only narrator is law enforcement	74 (48.7)	2 (5.0)	< 0.001
Number of gunshot wounds	70 (46.1)	5 (12.5)	< 0.001
Clinical condition of firearm-injured person	54 (35.5)	3 (7.5)	< 0.001
Relationship between firearm-injured person and shooter	24 (15.8)	1 (2.5)	0.03
Name of treating hospital	17 (11.2)	0 (0.0)	0.03
Video or audio depiction of shooting	12 (7.9)	2 (5.0)	0.03
Data or Epidemiological Trends, n (%)	18 (11.8)	15 (37.5)	< 0.001
Root Causes Presented, n (%)	8 (5.3)	13 (32.5)	< 0.001
Public health froot causes	4 (2.6)	11 (27.5)	< 0.001
Mention of prevention, mitigation and/or solutions, n (%)	15 (9.9)	25 (62.5)	< 0.001
Emphasis on prevention, n (%)			
No mention of prevention	137 (90.1)	11 (27.5)	< 0.001
Prevention is mentioned briefly	11 (7.2)	13 (32.5)	< 0.001
Prevention is a key focus	2 (1.3)	13 (32.5)	< 0.001
Specific solutions presented, n (%)	13 (8.6)	24 (60.0)	< 0.001
Resources offered to viewer, n (%)	0 (0.0)	4 (10.0)	0.28

*SD* Standard deviation

## Discussion

The results of this study offer important insights into local television news narratives on firearm violence in Philadelphia during the most significant surge in community firearm violence in US cities since the 1990s. We found that despite decades of evidence of the multilevel harms of episodic crime reports on violence, the majority of television news clips examined in this study contained episodic frames and featured law enforcement as the primary source of information [5, 7–9, 14, 16, 20–22]. There was limited humanizing information offered about the people who were shot, and episodic stories contained an average of 3 harmful content elements as identified by firearm-injured people [25]. One of our most significant findings is how rarely a public health perspective on firearm violence was presented. Evidence-based public health solutions to address firearm violence were seldomly featured in television news stories, and only 2% of clips offered resources for firearm violence prevention to news audiences. While thematic stories contained more public health narrators and solutions and less harmful content elements, they were not significantly longer than episodic stories. This finding indicates that thematic stories may be more feasible in the television news context than is traditionally thought by journalists. While it is likely that thematic stories may require more investigation time, increasing thematically framed stories on firearm violence could be an important first step in narrative change to center public health framing of this epidemic. More research is needed to understand how best to effectively engage news audiences with thematic stories that contextualize crime and violence, to ensure that these stories have the desired impact on viewers [20].

There is clear precedent for studies exploring harmful news content to inform journalistic policy development as a public health intervention. With empirical support, journalistic guidelines that provide special instruction to protect victims and audiences in cases of suicide, mass shootings, sexual assault, abuse, and crime involving minors have been widely accepted [38]. For example, research demonstrating that harmful reporting approaches are associated with increases in suicide incidence led to the adoption of revised newsroom practices endorsed by public health experts [39, 40]. These guidelines contain specific harm-reduction recommendations for news organizations, including avoiding: prominent story placement, sensationalizing headline/content, glamorization or oversimplification of suicide, discussing the suicide method, and repeated reporting about the same suicide [40]. They also advocate that journalists use language that is sensitive to the grieving family and provide resources for suicide prevention [40]. When news reports limit certain harmful approaches, portray suicide as preventable, and provide resources, studies have found that suicide rates decrease, a phenomenon called the Papageno Effect [41]. Importantly, no such guidelines crafted by journalists and public health practitioners exist for reporting on community firearm violence. The present study provides an important foundation on which to understand the elements of harm present in current news narratives on firearm violence. This research along with future work examining racial and place-based disparities in harmful reporting on community firearm violence could be used to inform guideline development for ethical reporting on community firearm violence.

Increasingly, professionals who care for and interact with survivors of firearm violence understand the need for trauma-informed approaches [42, 43]. Harmful reporting on community firearm violence that does not include the perspectives of firearm-injured people likely compounds trauma for survivors and co-victims of firearm violence [25]. The preponderance of potentially harmful content elements we found in television news stories about firearm violence indicates an urgent need to provide education to journalists in trauma-informed practices. Trauma-informed reporting on firearm violence would include journalists engaging with survivors using trauma-informed principles to minimize the distress of sharing their experiences and maximize survivors’ control over their injury narratives [44]. This type of reporting could humanize firearm-injured people and build empathy in audiences, deconstructing the existing racialized news narratives around firearm violence in cities. Reporting on community firearm violence that is trauma-informed should also minimize the elements that are known to cause harm to communities and society, including episodic crime frames, graphic visual imagery, and racialized narratives about the people and places impacted [5, 7–9, 14, 16, 20–22]. If sensationalized reporting on firearm violence is contributing to negative perceptions of public safety and in turn, increased rates of firearm purchasing as posited by some firearm-injured people, then addressing these harmful elements could actually contribute to firearm violence prevention [25].

Several of this study’s authors are engaged in work with The Philadelphia Center for Gun Violence Reporting (PCGVR), which is a community-based organization working to support ethical reporting on community firearm violence as a public health intervention [45]. Informed by this study and our previous research, PCGVR created and implemented a novel training course for journalists called *Gun Violence Prevention Reporting*. The goals of this program are to provide journalists with tools to employ trauma-informed practices when interacting with firearm violence survivors and co-victims and to create firearm violence stories that use thematic, solutions-oriented, and public health framing. Future research will include evaluation of the impact of this training program on news content and experiments to test the effects of thematic public health frames in stories on firearm violence on news audiences.

### Limitations

This study has limitations. While most of the clips included stories on community firearm violence events that occurred in Philadelphia, we did not characterize the etiology of firearm violence described in each clip due to challenges in doing so with limited information. In addition, this study includes local television news clips broadcast in a single city experiencing a surge in community firearm violence. Therefore, it may not be generalizable to other cities in the US, to national television news, or to print, radio, or social media content. In fact, it is possible that other television news stations across the US report firearm violence differently, which in turn could have an impact on community perceptions and responses to this public health problem. Examining news reports on firearm violence outside of Philadelphia will be a subject of future research. Finally, while television news is the legacy media source with the largest reach, more US adults get their news regularly from digital devices (56%) compared to television (32%) [29, 30]. Research that evaluates digital news content on firearm violence is needed to provide a complete picture of reporting on this public health issue.

## Conclusions

Community firearm violence is an increasing threat to public health in the United States. News reporting plays a critical role in educating audiences about the root causes and solutions to this epidemic. We found that most local television news stories on firearm violence in Philadelphia were episodic crime reports that contained multiple harmful elements as identified by firearm-injured people [25]. Local television news reports rarely framed firearm violence as a public health problem, resulting in innumerable missed opportunities to inform the public and policy makers on evidence-based public health interventions. Public health practitioners should partner with firearm violence survivors to offer alternative perspectives to journalists reporting on firearm violence. Journalists should engage in opportunities for training in trauma-informed reporting practices and solutions journalism. Importantly, newsrooms should adopt a public health approach to reporting on firearm violence that advances the idea that firearm violence is preventable, by offering solutions that are evidence-based, providing resources to audiences, and utilizing the key elements of a public health frame for reporting on firearm violence [8, 20, 23, 24].

### Supplementary Information

**Supplementary Material 1.**

**Supplementary Material 2.**

**Supplementary Material 3.**

## Data Availability

Television news content is available for download via subscription from TVEyes online (www.tveyes.com). The coded clip data created during the current study is available from the corresponding author on reasonable request.

## Abbreviations

US: United States
COVID-19: Coronavirus-19

## References

[1] Betz ME, Harkavy-Friedman J, Dreier FL, Pincus R, Ranney ML (2021). Talking about “firearm injury” and “gun violence”: words matter. Am J Public Health.

[2] Simon TR, Kegler SR, Zwald ML (2022). *Notes from th**e field* : increases in firearm homicide and suicide rates — United States, 2020–2021. MMWR Morb Mortal Wkly Rep.

[3] Schnippel K, Burd-Sharps S, Miller TR, Lawrence BA, Swedler DI (2021). Nonfatal firearm injuries by intent in the United States: 2016–2018 hospital discharge records from the healthcare cost and utilization project. West J Emerg Med.

[4] Leibbrand C, Hill H, Rowhani-Rahbar A, Rivara F (2020). Invisible wounds: community exposure to gun homicides and adolescents’ mental health and behavioral outcomes. SSM Popul Health.

[5] Iyengar S. Is anyone responsible? How television frames political issues. Chicago: Univ. of Chicago Press; 1994.

[6] Aubel AJ, Pallin R, Knoepke CE, Wintemute GJ, Kravitz-Wirtz N (2022). A comparative content analysis of newspaper coverage about extreme risk protection order policies in passing and non-passing US states. BMC Public Health.

[7] McKeever BW, Choi M, Walker D, McKeever R (2022). Gun violence as a public health issue: media advocacy, framing and implications for communication. Newsp Res J.

[8] Dorfman L, Woodruff K, Chavez V, Wallack L (1997). Youth and violence on local television news in California. Am J Public Health.

[9] Marvel D, Mejia P, Nixon L, Dorfman L. More than mass shootings: Gun violence narratives in California news. Issue, 25. 2018. p. 2–44. https://www.bmsg.org/resources/publications/gun-suicide-community-domestic-violence-news-narratives-california/.

[10] Pallin R, Aubel AJ, Knoepke CE, Pear VA, Wintemute GJ, Kravitz-Wirtz N (2021). News media coverage of extreme risk protection order policies surrounding the Parkland shooting: a mixed-methods analysis. BMC Public Health.

[11] Entman RM (1993). Framing: toward clarification of a fractured paradigm. J Commun.

[12] Kahneman D, Tversky A (1984). Choices, values, and frames. Am Psychol.

[13] Tuchman G. Making news: a study in the construction of reality. New York: Free Press; 1978.

[14] Thorson SRE (2001). The reporting of crime and violence in the Los Angeles times: is there a public health perspective?. J Health Commun.

[15] Graber DA. Crime news and the public. New York: Prager; 1980.

[16] Simon J, Hayes S (2004). Juvenile crime stories use police blotter without comment from suspects. Newsp Res J.

[17] Lipschultz JH, Hilt ML. Crime and local television news: dramatic, breaking, and live from the scene. L. Mahwah: Earlbaum Associates; 2002.

[18] Lowry DT, Nio TCJ, Leitner DW (2003). Setting the public fear agenda: a longitudinal analysis of network TV crime reporting, public perceptions of crime, and FBI crime statistics. J Commun.

[19] Bullock CF (2008). Official sources dominate domestic violence reporting. Newsp Res J.

[20] Coleman R, Thorson E (2002). The effects of news stories that put crime and violence into context: testing the public health model of reporting. J Health Commun.

[21] Parham-Payne W (2014). The role of the media in the disparate response to gun violence in America. J Black Stud.

[22] Callanan VJ, Rosenberger JS (2011). Media and public perceptions of the police: examining the impact of race and personal experience. Polic Soc.

[23] Dorfman L, Thorson E, Stevens JE (2001). Reporting on violence: bringing a public health perspective into the newsroom. Health Educ Behav.

[24] Coleman R, Perlmutter DD (2005). ‘Bullets as bacteria’: Television news magazines’ use of the public health model for reporting violence. Journalism.

[25] Beard JH, Midberry J, Afif IN, Dauer E, MacMillan J, Jacoby SF (2023). “Like I’m a nobody:” firearm-injured peoples’ perspectives on news media reporting about firearm violence. SSM Qual Res Health.

[26] Afif IN, Gobaud AN, Morrison CN (2022). The changing epidemiology of interpersonal firearm violence during the COVID-19 pandemic in Philadelphia, PA. Prev Med.

[27] Beard JH, Jacoby SF, Maher Z (2021). Changes in shooting incidence in Philadelphia, Pennsylvania, between March and November 2020. JAMA.

[28] Pino EC, Gebo E, Dugan E, Jay J (2022). Trends in violent penetrating injuries during the first year of the COVID-19 pandemic. JAMA Netw Open.

[29] Forman-Katz N, Matsa KE. News platform fact sheet. Pew Research Center’s Journalism Project. https://www.pewresearch.org/journalism/fact-sheet/news-platform-fact-sheet/. Accessed 17 Jan 2023.

[30] Jeon DS, Nasr N (2016). News aggregators and competition among newspapers on the internet. Am Econ J Microecon.

[31] Philadelphia-born action and eyewitness TV news format impacted Black America. https://www.inquirer.com/news/inq2/more-perfect-union-action-eyewitness-news-tv-racism-crime-20220329.html?outputType=default. Accessed 17 Jan 2023.

[32] Jacoby SF, Dong B, Beard JH, Wiebe DJ, Morrison CN (2018). The enduring impact of historical and structural racism on urban violence in Philadelphia. Soc Sci Med.

[33] Riffe D, Lacy S, Watson BR, Fico F (2019). Analyzing media messages: using quantitative content analysis in research.

[34] Dan V, D’Angelo P (2018). A methodological approach for integrative framing analysis of television news. Doing news framing analysis.

[35] Cassella K. Broadcast monitoring for TV and radio | TVEyes. TVEyes - search broadcast television and radio. https://www.tveyes.com/. Accessed 8 Sept 2023.

[36] Coleman R. Framing the pictures in our heads: exploring the framing and agenda-setting effects of visual images. In: Doing news framing analysis. New York: Routledge; 2009.

[37] Lacy S, Watson BR, Riffe D, Lovejoy J (2015). Issues and best practices in content analysis. Journal Mass Commun Q.

[38] SPJ Code of Ethics - Society of Professional Journalists. https://www.spj.org/ethicscode.asp. Accessed 17 Jan 2023.

[39] Niederkrotenthaler T, Braun M, Pirkis J, et al. Association between suicide reporting in the media and suicide: systematic review and meta-analysis. BMJ. Published online March 18, 2020:m575. 10.1136/bmj.m575. 10.1136/bmj.m575 PMC7190013 32188637

[40] Recommendations – reporting on suicide. https://reportingonsuicide.org/recommendations/. Accessed 17 Jan 2023.

[41] Till B, Arendt F, Scherr S, Niederkrotenthaler T. Effect of educative suicide prevention news articles featuring experts with vs without personal experience of suicidal ideation: a randomized controlled trial of the papageno effect. J Clin Psychiatry. 2018;80(1). 10.4088/JCP.17m11975. 10.4088/JCP.17m11975 30549483

[42] SAMHSA’s concept of trauma and guidance for a trauma-informed approach. Available: https://store.samhsa.gov/sites/default/files/sma14-4884.pdf.

[43] Timmer-Murillo SC, Schroeder ME, Trevino C (2023). Comprehensive framework of firearm violence survivor care: a review. JAMA Surg.

[44] Simpson R, Coté W. Covering violence: a guide to ethical reporting about victims and trauma, second edition. New York: Columbia University Press; 2006. p. 305.

[45] The Philadelphia Center for Gun Violence Reporting. The Philadelphia Center for Gun Violence Reporting. https://www.pcgvr.org/. Accessed 17 Jan 2023.

