# Supplementary material for: Public health framing of firearm violence on local television news in Philadelphia, PA, USA: a quantitative content analysis
Source: BMC Public Health. 2024 May 3;24:1221. doi: 10.1186/s12889-024-18718-0 (PMC11067069; doi:10.1186/s12889-024-18718-0)
Supplement: Supplementary file 1 — Supplementary Material 1. [file 12889_2024_18718_MOESM1_ESM.docx]

Additional File 1: Search Terms Used in the TVEyes Recording Program

| Search Terms | "gun violence" OR "gun law" OR "gun laws" OR "gun control" OR gun OR guns OR pistol OR handgun OR firearm OR revolver OR rifle OR "assault rifle" OR "automatic weapon" OR "automatic rifle" OR "assault rifle" OR "assault weapon" OR "semiautomatic" OR "semi-automatic" OR shot OR shots OR shoot OR shooting OR crossfire OR gunshot OR gunshots OR shooter OR bullet OR bullets OR fired OR "shell casing" OR "shell casings" OR homicide OR murder OR murdered OR killed OR wounded OR "crime scene" |
| --- | --- |
