# Supplementary material for: Public health framing of firearm violence on local television news in Philadelphia, PA, USA: a quantitative content analysis
Source: BMC Public Health. 2024 May 3;24:1221. doi: 10.1186/s12889-024-18718-0 (PMC11067069; doi:10.1186/s12889-024-18718-0)
Supplement: Supplementary file 2 — Supplementary Material 2. [file 12889_2024_18718_MOESM2_ESM.docx]

Additional File 2: Coded Variables with Intercoder Reliability Scores

| **Variable** | **Operationalization** | **Percent Agreement** | **Krippendorf’s Alpha** | **Gwet’s AC_1_** |
| --- | --- | --- | --- | --- |
| **Narrative Elements** |  |  |  |  |
| Framing | What is the primary frame of this story, based on time? (*Episodic, Thematic*)   - *Episodic*: majority of time spent discussing specific shooting event or shooting events - *Thematic*: majority of time spent discussing firearm violence more broadly, including social context, epidemiologic trends, root causes, and/or solutions | 97.8 | .921 | .969 |
| Narrators | Who is interviewed and/or shown speaking in the video aside from journalists? Check all that apply.  (*Law enforcement representative, Politician, Community representative, Firearm-injured person, Family member/friend/neighbor of firearm-injured person, Bystander/witness, Lawyer, Health professional, Unidentified person, Other, No one interviewed/shown other than journalist*) | 88.0 | .841 | .867 |
| Primary Narrator | Who is the primary narrator, defined by the person who is speaking/shown in video for the longest time?  (*Law enforcement representative, Politician, Community representative, Firearm-injured person, Family member/friend/neighbor of firearm-injured person, Bystander/witness, Lawyer, Health professional, Unidentified person, Other, No one interviewed/shown other than journalist*) | 86.7 | .813 | .854 |
| Police Attribution | Is some variation of "police say" or "according to police" mentioned? (*Yes, No*) | 93.3 | .867 | .868 |
| **Harmful Content Elements** |  |  |  |  |
| Visual Depiction of Crime Scene | Does the report include video or audio of a crime scene? (*Yes, No*) | 97.8 | .948 | .972 |
| Not a Follow-up Story | Is this a follow-up story? This is defined as a story that takes place more than 24 hours after a shooting event. (*Yes, No, Unknown, Not applicable*) | 75.6 | .588 | .696 |
| Number of Gunshot Wounds | Does the report include the number and/or location of gunshot wounds on firearm-injured person (shot once, shot multiple times, shot in the neck, etc.)? (*Yes, No*) | 95.6 | .878 | .946 |
| Clinical Condition of Firearm-injured Person | Does the report include description of the condition of the firearm-injured person (e.g. stable vs. unstable, critical, fighting for his life, going to be ok)? (*Yes, No*) | 95.6 | .907 | .942 |
| Relationship Between Firearm-injured Person and Shooter | Does the report include the relationship between firearm-injured person and shooter (including unknown)? (*Yes, No*) | 82.2 | .136 | .802 |
| Name of Treating Hospital | Does the report include the name of the treating hospital? (*Yes, No*) | 93.3 | .702 | .925 |
| Video or Audio of Shooting | Does the report include video or audio depiction of the shooting event? (*Yes, No*) | 100 | 1 | 1 |
| **Additional Visuals** |  |  |  |  |
| Police Imagery | Is police imagery (police cars, crime scene tape, crime scene footage (i.e. police are there, active crime scene), police officers in uniform, bullets, quotations from police expressed visually) shown in visual images? (*Yes, No*) | 93.3 | .733 | .911 |
| Photo or Interview with Firearm-injured Person and/or Family | Are photographs or interviews of the firearm-injured person /family/friends shown in visual images? (*Yes, No*) | 100 | 1 | 1 |
| Community Event | Are community events/activities (rallies, vigils, community organization activities) shown in visual images? (*Yes, No*) | 95.6 | .647 | .949 |
| Mugshot of Alleged Perpetrator | Are mugshots and/or photographs of the accused perpetrators shown in visual images? (*Yes, No*) | 91.1 | .457 | .894 |
| Political Press Conference | Do any visual images include a political press conference? (*Yes, No*) | 97.8 | .791 | .975 |
| **Characteristics of Firearm-Injured People** |  |  |  |  |
| Number of People Injured | How many victims are there? (Enter number) | 95.6 | .935 | .951 |
| Specific Firearm-injured person Identified | Does the reporting mention a specific shooting firearm-injured person? (*Yes, No*) | 97.8 | .934 | .967 |
| Fatality | Was the shooting fatal or non-fatal? *(Fatal, Non-fatal, Other)* | 97.1 | .945 | .962 |
| Age | What is the firearm-injured person’s age, if provided? (*Enter age, No age provided*) | 100 | 1 | 1 |
| Race/Ethnicity | What is the firearm-injured person’s race/ethnicity? (*White, Latine, Black, Asian, Mixed race/Multiracial, Unknown/Unclear from report*) | 97.1 | .912 | .966 |
| Gender | What is the firearm-injured person’s gender, if provided? (*Male, Female, Non-binary, Other, Unknown/Unclear from report*) | 97.1 | .921 | .964 |
| **Additional Personal Information** |  |  |  |  |
| Photograph | Is a photograph of the firearm-injured person shown? (*Yes, No*) | 100 | 1 | 1 |
| Name | What is the firearm-injured person’s name, if provided? (*Enter name, None provided*) | 100 | 1 | 1 |
| Family Information | Is information about the firearm-injured person’s family provided? (*Yes, No*) | 100 | 1 | 1 |
| Occupation | What is the firearm-injured person’s occupation? (*Enter occupation, None provided*) | 100 | 1 | 1 |
| Educational Level | What is the firearm-injured person’s educational level? (*Enter education level, None provided*) | 100 | 1 | 1 |
| Criminal Record | Is the criminal record of the firearm-injured person presented? (*Yes, No*) | 100 | 1 | 1 |
| **Elements of a Public Health Frame** |  |  |  |  |
| Epidemiological Context | Are data or epidemiologic trends mentioned? (*Yes, No*) | 95.6 | .810 | .942 |
| Root Causes | Does the story explore root causes of gun violence? (*Yes, No*) | 97.8 | .934 | .967 |
| Public Health Root Causes | What root causes of gun violence based on the public health approach are explored in the story? Check all that apply.  (*Poverty, Unemployment or lack of good quality employment, Food insecurity, Lack of quality education, Lack of opportunity, Covid-19 and containment policies, Housing inequality/red lining, Limited access to safe outdoor/green spaces, Police violence, Institutional violence, Mass incarceration, Mental health disorders (e.g. PTSD), Limited access to healthcare/health insurance, Limited access to mental health care, Racism, Increased or high levels of gun purchasing or gun ownership, Limited restrictive gun policies/permissive gun policies, Illegal gun use/ghost guns, Media coverage/fear, Drugs/opioid epidemic, Alcohol outlets/alcohol use, Other*) | 88.9 | .876 | .875 |
| Solutions | Does the story include any mention of gun violence prevention, mitigation, and/or solutions? (*Yes, No*) | 93.3 | .733 | .911 |
| Specific Solutions | Are specific recommendations made for interventions to prevent or mitigate gun violence? (*Yes, No*) | 90.9 | .784 | .841 |
| Public Health Solutions | What public health prevention interventions are discussed? Check all that apply.  (Increased restrictive gun policies, Decreased permissive gun policies, Gun buy-back programs, Increased access to high-quality education, Increased access to high-quality housing, Increased access to safe green/outdoor spaces, Increased minimum wage/decrease in pay gap, Social services, Increased access to healthcare, Increased access to mental healthcare, Increased access to employment/better jobs, Hospital-based violence intervention programs, Cure violence/Community violence interruption, Better reporting, Increased research volume and/or funding, Gun locks, Safe storage, Conflict resolution, Individual mental health care, Mentorship, Smaller-scale community programs, Other) | 100 | 1 | 1 |
| Emphasis on Prevention | Does the story emphasize prevention? That is, is prevention a key focus or is it just mentioned briefly? (*Prevention is a key focus, Prevention is mentioned briefly, No mention of prevention*) | 86.7 | .409 | .856 |
| Includes Word Prevent | Does the story include the words “prevent” and/or “prevention” in reference to gun violence? (*Yes, No*) | 100 | 1 | 1 |
| Resources Offered | Are any resources offered to the viewer? (*Yes, No*) | 100 | 1 | 1 |
