# Supplementary material for: Public health framing of firearm violence on local television news in Philadelphia, PA, USA: a quantitative content analysis
Source: BMC Public Health. 2024 May 3;24:1221. doi: 10.1186/s12889-024-18718-0 (PMC11067069; doi:10.1186/s12889-024-18718-0)
Supplement: Supplementary file 3 — Supplementary Material 3. [file 12889_2024_18718_MOESM3_ESM.docx]

Additional File 3: Text from Exemplar Philadelphia Television News Stories on Firearm Violence, January – June, 2021

Exemplar 1: Episodic report with police attribution: NBC 10 on February 7, 2021 at 11:00 p.m.

Journalist: “Back here at home, gunfire inside of a local mall. A person is hurt and another is on the run following a shooting inside the Philadelphia Mills Mall, formerly Franklin Mills Mall. Police say somebody opened fire just before 1:30 this afternoon. This video shows officers in the parking lot. Inside, a 21-year-old man was shot in the neck. He is expected to be okay. Investigators are still looking for the shooter.”

Exemplar 2: Episodic report with law enforcement representative as primary narrator: CBS 3 on April 15, 2021 at 6:00 a.m.

Journalist: “Good morning, and even before this latest deadly shooting in the city of Philadelphia, police here had already recorded 142 homicides, this year alone, and that's a 33% increase from this time last year. Let's talk about this specific case though. That six-year-old little boy who was shot and injured is currently in critical but stable condition this morning. His 28-year-old father, however, was shot and killed, and a 30-year-old man was also shot and injured during a hail of gunfire last night in Southwest Philadelphia. So, let's take you to the scene now tell you what we know so far about this shooting. It all unfolded just after 7:00 near X street. Police say those two men and that six-year-old little boy were sitting in a parked car, when someone opened fire, shooting at least 12 times. Now the 28-year-old man who was in the driver’s seat was shot twice in the chest. Police say he tried to drive away but eventually crashed into several parked cars. Meantime, that man's six-year-old son was shot in his back and stomach, and a 30-year-old man was shot once in the chest. Police say he was actually able to get away and flag down police for help two blocks from the shooting scene at X Avenue. Now, all three victims were taken to the hospital, and that's where the 28-year-old was pronounced dead. Police are now looking for the gunman as well as the motive for this attack. Police Commissioner, Danielle Outlaw, says it is still unclear if the gunman knew there was child in that car, and she says she is fed up with responding to scenes just like this one.” Outlaw: “Just, you know, couple of days ago we saw a nine-year-old who fortunately, the nine-year-old was only shot in the knee. But we all got to do better with ensuring that our most vulnerable including our young people are safe. This includes our adults, ensuring that our young people in our care and entrusted in our care are safe, and that's not just those who are parents. Family members that have young people with them, but those who are committing these acts of violence-just sickens me, sickens all of us. We're tired of seeing this, quite frankly.” Journalist: “Police are looking for surveillance video as well as looking for eyewitness that may be able to help. Police do believe the gunmen were in a white colored car with heavily tinted windows. Anyone with information should call police. Reporting live outside Philadelphia Police headquarters.”

Exemplar 3: Thematic report with community members as narrators: FOX 29 on April 15, 2021 at 11:00 p.m.

Journalist: “We know you're tired about hearing about all of the shootings, and we are tired of reporting on them, but kids are dying at a heartbreaking pace we cannot ignore. It is only April and so far 50 kids have been shot. Fifteen of them shot and killed in Philadelphia. Where is the outrage? We had three very young victims this week alone. A 9-year-old shot in his home, 16-year-old gunned down in the street, and 6-year-old shot while sitting in a car with his dad. Developing tonight, it happened again, two teens shot after leaving a rec center in a possible drive-by. This happened it happened in South Philly. A 17 and 18-year-old hit in the foot and leg. We are told they will be okay, but this violence is clearly out of control. XX reports, some are trying to tackle the problem one piece at a time.” Journalist: “Many families showed up with young people to help register them for a summer job opportunity. In the video, you will see a few people inside at a time due to Covid precautions, but this is how the community is trying to curb violence.” Community member: “I skateboard every day so I have something to do.” Journalist: “Fourteen-year-old XX stays productive doing something he loves, skateboarding. He is making plans for the future to buy a car when he turns 18.” Community member: “I wanted a job to make extra money.” Journalist: “Today, he took one step toward the goal, showing up this evening at X community center in South Philly for summer youth employment events.” Community member: “It is out of control and we need help in this city because these young men are killing each other, wounding innocent bystanders.” Journalist: “XX's grandparents brought him here out of concern for what they see happening on the streets of Philadelphia with other young people.” Community member: “First thing they think they can do is make money on the corner with drugs. someone comes into their territory, that is when the violence starts.” Journalist: “The violence XX says is largely due to lack of resources and employment.” Community member: “It’s big part of the problem, if you teach them young what it means to get up and work and do something positive, they will carry it out through their lives.” Journalist: “X is the founder of Unity in the Community. Community member: “Kids getting killed. It is up to us as leaders to create opportunities for them.” Journalist: “Executive director of the NOMO foundation which stands for New Options More Opportunities has worked with youth in poverty and against violence. In his experience, young people want better.” Community member: “Most kids that find themselves on the corner don’t just find themselves on the corner. They are recruited to the corner due to their situation at home. A lot of kids really want out. They don’t really want to be there. We create environments like this and spaces like this and they flock to it.” Journalist: “Organizers say people who don't have parents or mentors to bring them to events like this, they plan on going door-to-door into communities and posting on social media. If you would like more information on these summer job opportunities for young people just go to fox29.com.”
